# Evaluation of tumor M2-pyruvate kinase (Tumor M2-PK) as a biomarker for pancreatic cancer

**DOI:** 10.1186/s12957-018-1360-3

**Published:** 2018-03-14

**Authors:** Indika A. Bandara, Minas Baltatzis, Sudip Sanyal, Ajith K. Siriwardena

**Affiliations:** 1 Regional Hepato-Pancreato-Biliary Unit, Manchester Royal Infirmary, Oxford Road, Manchester, M13 9WL UK; 2 Faculty of Biology, Medicine and Health, University of Manchester, Manchester, UK

**Keywords:** Pancreatic cancer, Cancer prognosis, Tumor M2-PK

## Abstract

**Background:**

Expression of the dimeric M2 isoenzyme of pyruvate kinase, termed Tumor M2-PK, is increased in some human cancers. This study evaluates the potential role of pre-operative Tumor M2-PK as a marker of prognosis in patients with pancreatic malignancy.

**Methods:**

Seventy-three consecutive patients with a clinical diagnosis of pancreatic or peri-ampullary cancer were enrolled. Their median (range) age was 66 (23–83) years. Pre-operative samples of venous blood were taken for analysis of Tumor M2-PK. The full study protocol was approved by the North West Research Ethics Committee (protocol number 06/MRE08/69).

**Results:**

The mean (standard deviation) plasma Tumor M2-PK in pancreatic/peri-ampullary malignancy was 60.3 (106.5) U/ml and 22 U/ml (SD: 12 U/ml) in benign disease (*p* < 0.001). Multivariate Cox regression analysis showed that Tumor M2-PK (> 27 U/mL), Ca19-9 (> 39 U/ml), resection status, and disease stage were associated with poorer survival. Tumor M2-PK values greater than 27 U/ml were associated with inferior survival compared to those with lower values (hazard ratio 2.049, significantly increased risk of death, *p* = 0.042).

**Conclusion:**

This preliminary study shows that an elevated level of Tumor M2-PK (with a cutoff threshold of 27 U/mL) measured pre-operatively is associated with poorer prognosis in patients with pancreatic and peri-ampullary cancer.

**Electronic supplementary material:**

The online version of this article (10.1186/s12957-018-1360-3) contains supplementary material, which is available to authorized users.

## Background

In mammalian tissues, there are four different molecular isoforms of pyruvate kinase [1–3]. The dimeric M2 isoenzyme is over-expressed in a range of cancers [4–9]. Using an enzyme-linked immunosorbent assay (ELISA) assay, we previously evaluated the role of tumor M2-pyruvate kinase as a diagnostic test for pancreatic cancer [10]. The mean (SD [standard deviation]) plasma Tumor M2-PK level for patients with histologically proven malignancy was 40.5 (26.4) U/mL and for non-cancer patients, 29.9 (20.9) U/mL (Mann-Whitney *U* = 1163, *p* = 0.006) [10]. Tumor M2-pyruvate kinase had an area under the curve (AUC) of 0.623 on receiver operating characteristic curve analysis and at an optimal cutoff of 27 U/mL, sensitivity was 66%, and specificity was 58%. On multivariate Cox regression modeling, elevated Tumor M2-PK (> 27 U/mL) was strongly correlated with the subsequent finding of poorly differentiated cancer and/or metastatic disease and strongly predicted adverse survival on Kaplan-Meier analysis. The diagnostic accuracy of Tumor M2-PK was not affected by jaundice. These findings suggest that although the test is unlikely to be of value as a diagnostic aid, there may be a potential niche as an indicator of prognosis. The present study assesses the prognostic value of Tumor M2-PK in pancreatic and peri-ampullary cancer.

## Methods

### Study design

This is a single center, prospective cohort study comparing pre-operative plasma Tumor M2-PK measurement to outcome in patients with suspected pancreatic/peri-ampullary cancer.

### Setting

The study was conducted in the regional Hepatobiliary and Pancreatic (HPB) cancer service of the Manchester Royal Infirmary. This is a tertiary HPB service treating a predominantly urban conurbation of 3.2 million.

### Patients

Patients referred with a clinical diagnosis of pancreatic or peri-ampullary cancer between May 2011 and February 2014 constituted the study population. Patients being staged for resection of pancreatic tumors were included if they were older than 18 years of age and if there was no evidence of metastatic disease. Only patients able to give full informed consent were included. Exclusion criteria included inability to give informed consent, age younger than 18 years and a histologically confirmed concurrent non-pancreatic malignancy or a subsequent diagnosis of a non-pancreatic malignancy during the follow-up period. Those who were confirmed to have malignancy from pre-operative cytology as well as patients with only a clinical/radiological diagnosis of pancreatic cancer confirmed by the Multidisciplinary Team (MDT) were included in the survival analysis, whereas patients with histologically confirmed benign lesions after resection were excluded. Seventy-three consecutive patients with a clinical diagnosis of pancreatic or peri-ampullary cancer were enrolled. Their median (range) age was 66 (23–83) years. Eight patients (11%) were diagnosed as having unresectable disease pre-operatively during work-up and did not undergo surgical treatment. Of the 65 (89% of the entire cohort) who underwent surgical treatment, 55 had resection of their tumor and 10 had palliative non-resectional treatment. Nine patients (12%) undergoing resection of suspected malignant disease had benign histology on resection and were excluded from the survival analysis.

### Sample collection, storage, and biochemical assays

Five milliliter of whole venous blood was collected into an EDTA (ethylene diamine tetra-acetic acid) bottle at room temperature. The sample was spun for 10 min at 3000 rpm within 12 h of collection. Plasma was separated into two aliquots in Z5 screw top tubes and stored immediately at − 80 °C. The final batch analysis for Tumor M2-PK was performed at the ScheBo Biotech laboratory (Geissen, Germany) in compliance with previously published protocols [10]. CA 19-9 analysis for this study was undertaken as part of routine clinical care by the National Health Service reference laboratory at the Christie Hospital, Manchester.

### Data collection

Electronic case report forms were used to collect information on the following: demographic data: Age, gender, smoking status, co-morbidities; clinical data on presentation, results of hematological, biochemical, radiological, cytological investigations; and operative detail in those patients undergoing surgery and histology together with peri-operative outcome, survival, and follow-up data.

### Data analysis

The Statistical Package for the Social Sciences version 21.0 (IBM Corp; IBM SPSS Statistics for Windows, Version 21.0, Armonk, NY, USA) was utilized for statistical analysis. Receiver operator curves were generated for Tumor M2-PK. Multivariate Cox regression was used for survival analyses. The Kolmogorov-Smirnov test was used to assess for parametric (normal) distribution and two sample comparisons were by *t* test. A probability of less than 0.05 was considered statistically significant.

### Ethics committee approval

The study was approved by the North West Regional Ethics Committee (reference number 10/H1014/86). All patients gave their written informed consent for participation.

## Results

### Patients

Demographic and disease profiles are seen in Table 1.

**Table 1:** Demographic and disease profile

Age (median––range)	66 (23–83)
Gender	Male 52 (71%) Female 21 (29%)
Management	
Surgical treatment	*65 (89%)*
Resection	*55*
Pancreaticoduodenectomy	47
Distal pancreatectomy + splenectomy	5
Total pancreatectomy	2
Central pancreatectomy	1
No resection	*10*
Palliative procedures	8
Intraoperative biopsy only	2
No surgical treatment	*8 (11%)*
Histology of resected specimen	
Malignant	*46 (63%)*
Pancreatic ductal adenocarcinoma	18
Ampullary adenocarcinoma	15
Neuroendocrine tumor	6
Distal bile duct cholangiocarcinoma	4
Duodenal adenocarcinoma	2
Pancreatic adenosquamous carcinoma	1
Benign	*9 (12%)*
Groove pancreatitis	2
Chronic pancreatitis	2
Ampullary tubulovillous adenoma	2
Mucinous neoplasm	1
Serous cystadenoma	1
Solid pseudopapillary tumor	1
Histology/cytology from biopsy	*18 (25%)*
Pancreatic ductal/ampullary adenocarcinoma	8
Neuroendocrine tumor	1
Adenocarcinoma (frozen section)	5
Histology not confirmed (clinical/radiological diagnosis)	4
Cancer differentiation^a^	
Well and moderately differentiated	34
Poorly differentiated	16
Resection margin^b^	
R0	18
R1	28

*R0* complete histological resection with at least 1-mm clear resection margin. *R1* tumor reaches surgical resection margin

^a^Available for 46 patients who underwent resection and a further 4 who underwent biopsy

^b^Applies only to patients undergoing surgical resection for cancer

The values in italics denote statistical significance

### Plasma Tumor M2-PK and CA 19-9

In patients with pancreatic/peri-ampullary cancer, Tumor M2-PK 60.3 (SD 106.5) U/ml compared to 22 (SD 12) U/ml (SD 12 U/ml) in patients without malignancy (*p* < 0.001). Serum Ca19-9 was 1750 (SD 4817) U/ml in those with pancreatic/peri-ampullary cancer and 16 (12) U/ml in patients without malignancy (*p* = 0.004).

### Tumor M2-PK as a predictor of survival

**Fig. 1:**
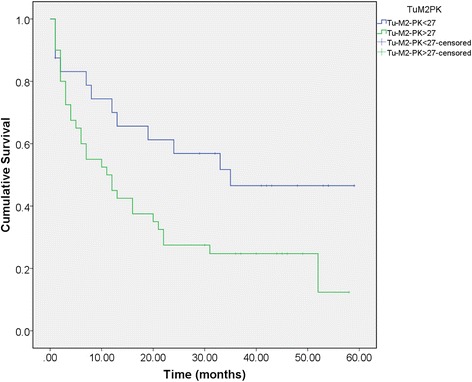
Kaplan-Meier survival plot. Survival in months from the time of presentation according to Tumor M2-PK greater or lower than 27 U/mL

**Fig. 2:**
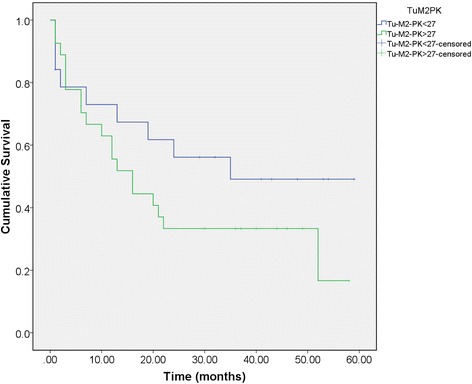
Kaplan-Meier survival plot for patients undergoing surgical resection

Tumor M2-PK (> 27 U/mL), Ca19-9 (39 U/ml), resection status (resection or not), and advanced stage (stages III and IV) correlated with inferior survival. Patients with Tumor M2-PK higher than 27 U/ml had worse prognosis than those with lower values (hazard ratio 2.049, significantly increased risk of death, *p* = 0.042). Figure 1 shows the Kaplan-Meier survival curves for Tumor M2-PK above and below the cutoff level. Figure 2 shows the Kaplan-Meier curves in the subgroup of patients who underwent surgical resection.

### Pre-operative Tumor M2-PK as predictor of advanced stage

Two separate ROC curve analyses were performed to assess sensitivity and specificity of Tumor M2-PK in predicting advanced disease stage (III and IV) and tumor unresectability, respectively. Tumor M2-PK at a cutoff value of 25.37 U/ml had a sensitivity of 85% and specificity of 43% in predicting stage III/IV disease (Table 2). The negative predictive value (NPV) was 74% and positive predictive value (PPV) 60%. Area under curve (AUC) was 0.583. Tumor M2-PK cutoff value of 27.82 U/ml had a sensitivity of 72% and specificity of 42% in predicting unresectability. AUC was 0.514, NPV = 60%, and PPV = 55%.

**Table 2:** Exploratory survival analysis

	*b*	SE	Wald	dF	Sig	Exp(B) (hazard ratio)
Base model						
Age	0.010	0.014	0.563	1	0.453	1.010
Gender	− 0.167	0.327	0.261	1	0.609	0.846
Incremental model: tumor M2-PK > 27 U/mL						
Age	0.004	0.015	0.076	1	0.782	1.004
Gender	− 0.221	0.329	0.452	1	0.501	0.802
Tumor M2-PK > 27 U/ml	0.718	0.353	4.132	1	*0.042*	2.049
Incremental model: Ca 19-9> 39						
Age	0.010	0.014	0.472	1	0.492	1.010
Gender	− 0.515	0.351	2.153	1	0.142	0.598
Ca 19-9> 39	1.081	0.345	9.821	1	*0.002*	2.949
Incremental model: resection						
Age	0.013	0.014	0.862	1	0.353	1.013
Gender	− 0.233	0.330	0.501	1	0.479	0.792
Resection	− 0.632	0.326	3.744	1	0.053	0.532
Incremental model: staging						
Age	0.014	0.015	0.797	1	0.372	1.014
Gender	− 0.250	0.331	0.569	1	0.450	0.779
Stage I	− 1.191	0.628	3.594	1	0.058	0.304
Stage II	− 0.529	0.318	2.762	1	0.097	0.589
Stage III + IV	0.635	0.308	4.261	1	*0.039*	1.888

*b* regression coefficient, *SE* standard error of regression coefficient b. *Wald* Wald statistic (b/SE)2, *Df* degrees of freedom, *Exp (B)* hazard risk ratio

The values in italics denote statistical significance

## Discussion

This study has re-evaluated plasma Tumor M2-PK as a potential marker of prognosis in patients with pancreatic and peri-ampullary cancer. As the dimeric M2 isoform detected by the assay is overexpressed in cancer, the hypothesis that patients with a poorer prognosis cancer are more likely to have disseminated disease and thus more sources of Tumor M2-PK production seems reasonable [11, 12]. Our previous study showed that Tumor M2-PK, unlike CA 19-9 was not affected by pre-operative jaundice but was insufficiently accurate for use as a diagnostic test. An interesting finding of that study was that there was differential survival according to pre-operative Tumor M2-PK with patients having values above 27 U/mL having worse outcome compared to those with values lower than this. The present study sets out to explore this phenomenon.

In relation to the present study, although this was carried out in a regional hepato-pancreato-biliary center, it is acknowledged that the sample number is small, that during the period of the study, there was a surprisingly high proportion of patients undergoing resection for suspected malignancy with a final diagnosis of non-cancer and a small proportion of patients included in the analyses had clinical and radiological diagnoses of pancreatic malignancy but did not have tissue confirmation of cancer. A protocol which included serial post-operative measurement would also have helped to assess whether resection of macroscopic disease with clear resection margins was associated with a fall in Tumor M2-PK.

Accepting these limitations, there are some important findings from this study. First, the study confirms again that Tumor M2-PK is elevated in patients with pancreatic and peri-ampullary malignancy compared to patients without cancer. Although our previous study sought to utilize the diagnostic potential of this test by using it in combination with CA 19-9, current information suggests that Tumor M2-PK is not sufficiently accurate for use as a diagnostic test. When measured pre-operatively, the test does appear to have discriminant value in terms of predicting prognosis with values above 27 U/mL being associated with shortened survival. Currently, the EDTA plasma test for Tumor M2-PK is undertaken as an immune-assay of stored batched samples. If further studies indicate a viable clinical role for this assay, single-sample analytic techniques will be required.

Both the present study and our previous report are relatively small studies, and thus, the findings are insufficiently robust to recommend general adoption of the test without further confirmation. Pooling of the results of the two studies would be unwise as the samples were from widely disparate time periods. Other studies do also support an association between elevated Tumor M2-PK and poor prognosis [13, 14]. Future work could also examine the role of Tumor M2-PK as a component of a diagnostic and prognostic panel in combination with other markers such as D-Dimer which are differentially expressed in pancreatic cancer [15].

## Conclusion

In conclusion, this study has examined the role of Tumor M2-PK as a marker of prognosis in patients with pancreatic and peri-ampullary cancer. The findings show that a value in excess of 27 U/mL is associated with shortened survival.

## Additional file

Additional file 1: The original dataset is provided as a supplementary file. (XLSX 21 kb)
